# Multi-Agent Hierarchical Graph Attention Actor–Critic Reinforcement Learning

**DOI:** 10.3390/e27010004

**Published:** 2024-12-25

**Authors:** Tongyue Li, Dianxi Shi, Songchang Jin, Zhen Wang, Huanhuan Yang, Yang Chen

**Affiliations:** 1 Academy of Military Sciences, Beijing 100097, China; 2 Tianjin Artificial Intelligence Innovation Center (TAIIC), Tianjin 300450, China; 3 College of Computer, National University of Defense Technology, Changsha 410073, China; 4 School of Computer Science, Peking University, Beijing 100871, China

**Keywords:** hierarchical graph attention, multi-agent reinforcement learning, curriculum learning

## Abstract

Multi-agent systems often face challenges such as elevated communication demands, intricate interactions, and difficulties in transferability. To address the issues of complex information interaction and model scalability, we propose an innovative hierarchical graph attention actor–critic reinforcement learning method. This method naturally models the interactions within a multi-agent system as a graph, employing hierarchical graph attention to capture the complex cooperative and competitive relationships among agents, thereby enhancing their adaptability to dynamic environments. Specifically, graph neural networks encode agent observations as single feature-embedding vectors, maintaining a constant dimensionality irrespective of the number of agents, which improves model scalability. Through the “inter-agent” and “inter-group” attention layers, the embedding vector of each agent is updated into an information-condensed and contextualized state representation, which extracts state-dependent relationships between agents and model interactions at both individual and group levels. We conducted experiments across several multi-agent tasks to assess our proposed method’s effectiveness, stability, and scalability. Furthermore, to enhance the applicability of our method in large-scale tasks, we tested and validated its performance within a curriculum learning training framework, thereby enhancing its transferability.

## 1. Introduction

Multi-agent systems have attracted substantial research attention due to their widespread presence and crucial roles in various domains. In nature, these systems are evident in ecosystems and food chains [1,2]. In industrial applications, they are indispensable in automated manufacturing and robotics [3,4], intelligent transportation [5], coordinated patrol [6], formation control [7], and cooperative navigation [8]. Interactions within multi-agent systems are broadly categorized into cooperative and competitive relationships. Cooperative interactions involve agents working together to achieve shared goals, while competitive interactions imply that agents compete for resources to fulfil individual objectives [9]. Additionally, mixed cooperative–competitive relationships are common in many fields, exemplified by predation among animal groups and symbiotic relationships among fish [10,11]. However, as multi-agent systems are increasingly applied to more complex tasks with a growing number of agents, interaction information between agents is becoming more and more frequent. Agents need to interact with important targets in a dynamic environment to acquire crucial information, which enables them to make informed decisions.

Multi-agent reinforcement Learning (MARL) is a crucial approach for addressing collaboration or competition problems in multi-agent systems [12,13]. Previous MARL research has predominantly focused on fully cooperative strategies, aiming to maximize the collective reward of agents, which is suitable for fully cooperative tasks. However, in both natural and industrial applications, mixed cooperative–competitive relationships are common and widespread, such as in multi-UAV pursuit and missile interception tasks [14,15,16,17,18]. In mixed cooperative–competitive tasks, cooperation and competition are often interwoven. The teammates collaborate to achieve common goals while simultaneously competing against opponents to pursue individual or subsystem interests [19]. Consequently, most fully cooperative MARL methods are inadequate for addressing these complex scenarios. Additionally, as multi-agent systems are increasingly applied to more complex tasks with a growing number of agents, large-scale modelling inevitably leads to the curse of dimensionality, greatly complicating training processes. A more fundamental challenge is the limited transferability of these models. Previous MARL methods have rarely considered strategy transferability, limiting their application to tasks with variable agent numbers and hindering their effectiveness in complex multi-agent tasks.

Multi-agent interactions can be naturally modeled as a graph, where nodes represent agents and edges represent their interactions [20]. Agents can share crucial information through connections to enable mutual learning and strategy optimization. However, as the frequency of agent interactions rises, information complexity escalates. Full graph connections and global information exchange are costly and can lead to information redundancy.

In practice, most real-world environments exhibit partial observability and limited communication (due to limited range or noisy sensors) [21]. Agents usually communicate only with neighbouring agents to reduce communication costs, meaning they must learn to collaborate based on local observations and limited communication. In multi-agent systems, where nearby agents have a more significant influence on each other’s behaviour, agents should wisely choose whom to communicate with, bake key information into the graph architecture, and transmit valuable information to appropriate teammates to facilitate collaboration and learn more efficient strategies [22].

Motivated by the aforementioned discussions, we propose a multi-agent hierarchical graph attention actor–critic reinforcement learning method (MAHGAC). We model the multi-agent interactions as a graph and use a hierarchical graph attention mechanism (HGAT) to encode the local observations of each agent as a single node embedding vector. This vector encapsulates an information-condensed and contextualized state representation, aggregating the state dependencies among agents and capturing both individual and hierarchical relationships. Therefore, agents can learn to assign importance weights to neighbouring agents, dynamically select optimal teammates for cooperation or opponents for focus, learn more advanced strategies, and improve the performance of multi-agent systems in complex interactions. In summary, our work makes the following contributions:

- Multi-agent interactions are effectively modelled as graphs, where agents are represented as nodes, and their connections form edges through which information is exchanged. Graph attention networks encode each agent’s local observations into a single node embedding vector. The dimensionality of this embedding vector remains constant regardless of the number of agents and generates a fixed-size environment representation, offering flexibility and scalability.
- We propose a hierarchical graph attention mechanism to optimize the efficient information extraction of agents in complex environments. The HGAT transforms the agents’ observation information into an information-condensed and contextualized state representation to capture relationships at both individual and hierarchical levels using the “inter-agent” and “inter-group” attention layers. By aggregating individual and hierarchical relationships, agents can better “understand” the dynamic environment changes, focus on interacting with the most relevant agents, and thus learn more “advanced” strategies.
- To validate the transferability of our method, we trained it within a curriculum learning framework. With curriculum learning, agents can gradually adapt to new tasks with varying numbers of agents, enabling the trained strategies to be effectively transferred to new tasks, thereby enhancing their transferability. Using curriculum learning, we successfully transferred a five-agent line formation strategy to a new task with ten agents.

The rest of this paper is structured as follows: Section 2 reviews existing works. Section 3 presents the problem formulation and relevant preliminary knowledge. Section 4 details the methodology, including mathematical formulations and the algorithm’s training procedure. Section 5 outlines the experimental environment setup, baseline methods, and evaluation metrics and provides an analysis of the experimental results. Section 6 presents the transferability within the curriculum learning framework. Finally, Section 7 summarizes the conclusions.

## 2. Related Works

In multi-agent systems, agents must observe environmental information and exchange critical knowledge with one another to coordinate actions and achieve collective goals. Given the enormous volume of information, agents must determine which peers to prioritize in information sharing to foster cooperation and enhance overall system performance. Information acquisition and transfer among agents are crucial for effective strategy learning.

Recent research has increasingly employed attention mechanisms in multi-agent systems. Ma et al. [23] propose a novel interactive advantage actor–critic collaborative MARL method (IAC). The method utilizes a shared attention mechanism to evaluate the functions of each agent in value functions, considering the influence of teammates. Iqbal et al. [24] propose an actor–attention–critic (MAAC) MARL method, which uses an attention mechanism to select the relevant information for each agent at each step. The mechanism functions similarly to a differentiable key-value memory model, where each agent queries the currently relevant observational and action information from other agents and integrates this information into the value function estimation, computing the agent’s gradient estimate.

Modelling multi-agent systems such as graphs with graph attention mechanisms has become a key method for solving information interactions between agents. Su et al. [25] presents an agent communication architecture that employs graph convolution to represent agent communication, where the flexibility of the graph structure enables the method to be applied to a variety of multi-agent systems. Liu et al. [26] propose a two-stage graph attention network (G2ANet). It establishes relationships between agents through a complete graph, utilizing soft and hard attention mechanisms to learn whether interactions exist between two agents and learn the importance of these interactions. Jiang et al. [27] propose a graph convolutional reinforcement learning (DGN) method, which adapts to the dynamics of the underlying graph of the multi-agent environment and learns cooperation using latent features through the convolutional layers with a gradually increased receptive field, and cooperation is further enhanced through temporal relationship regularization. Sun et al. [28] propose a multi-attention interaction modelling method (IMMA), utilizing multiple latent graphs to represent the interactions and attention of various independent types, thereby considering different strength relationships among agents. Niu et al. [22] propose a multi-agent graph attention communication (MAGIC), which uses the graph attention communication protocol to allow agents to learn when to communicate and to whom they send information to.

In multi-agent systems, agents must prioritize peers for information sharing to foster cooperation and enhance overall system performance. Attention mechanisms are an effective method of optimizing information interactions between agents. However, as task difficulty and complexity increase, the volume and complexity of information exchanged between agents also grow. Agents must extract critical information from vast, dynamically changing environments and determine state dependencies between agents to facilitate more effective collaboration.

## 3. Preliminaries

### 3.1. Partially Observable Markov Game (POMG)

**Figure 1:**
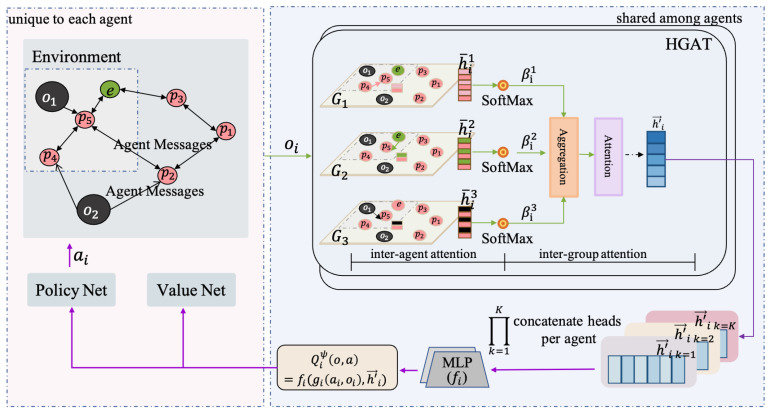
The overall structure of the MAHGAC. Left: an interactive multi-agent pursuit environment. Right: a shared HGAT module. The MAHGAC adopts the centralized training and decentralized execution (CTDE) training paradigm. During the training, adopting a centralized critic and sharing a hierarchical graph attention mechanism, agent *i* can obtain information from all agents and learn the importance weights of other agents in its vicinity. During the testing, each agent executes actions based on its own observations.

We abstracted multi-agent problem as a partially observable Markov game (POMG) based on the observational capabilities and dynamic attributes of *N* agents: s∈S denotes the global state of the game. $a_{i}\in A_{i}$ is an action for agent *i*. State transition function defines the probability distribution of the agent transitioning to the next state based on the current actions of each agent: $S\times A_{1}\times \cdots \times A_{N}⟶P\left(S\right)$. The reward for agent *i* is computed as a function of state *s* and joint action *a* as: $R_{i}:S\times A_{1}\times \cdots \times A_{N}⟶R$. $o_{i}\in O_{i}$ denotes a local observation of agent *i*, which contains partial information from the global state s∈S. As shown in Figure 1, in the multi-agent pursuit environment, the observable space of the agent *i* includes the location information and velocity information of entities within the observation range V(i):$o_{i}=\left\{s_{j}|j\in V\left(i\right)\right\}$. For pursuer $p_{5}$, the observation information is: the position $p_{4}:\left(p_{x4}^{p},p_{y4}^{p}\right)$, the velocity $p_{4}:\left(v_{x4}^{p},v_{y4}^{p}\right)$, the position of prey $e:(p_{x}^{e},p_{y}^{e})$, the velocity of prey $e:(v_{x}^{e},v_{y}^{e})$, and the position of obstacle $o_{1}:\left(p_{x1}^{o},p_{y1}^{o}\right)$. The action space *A* is the speed of the agent at the next time $\left(v_{x},v_{y}\right)$. Each agent learns a strategy $\pi_{i}:O_{i}\to P\left(A_{i}\right)$. The agent *i* aims to maximize its discounted return: $R_{i}=\sum_{t=0}^{T}\gamma^{t}r_{i}^{t}$, where γ∈[0,1] is a discount factor.

### 3.2. Graph Attention Network (GAT)

The graph attention network (GAT) is an effective model to process structured data that are represented as a graph. GAT proposes a way to calculate the target node embedding vector ${\vec{h}}_{i}^{\prime }=\sigma \left(\sum_{j\in N_{j}}\alpha_{ij}W_{{\vec{h}}_{j}}\right)$ of the graph by aggregating the node embedding vectors ${\vec{h}}_{j}$ from the neighbour nodes $\left\{j\in N_{i}\right\}$ that are connected with the target node *i*. The GAT uses self-attention to aggregate information from neighbouring nodes to adaptively match weights for different neighbours.

**Figure 2:**
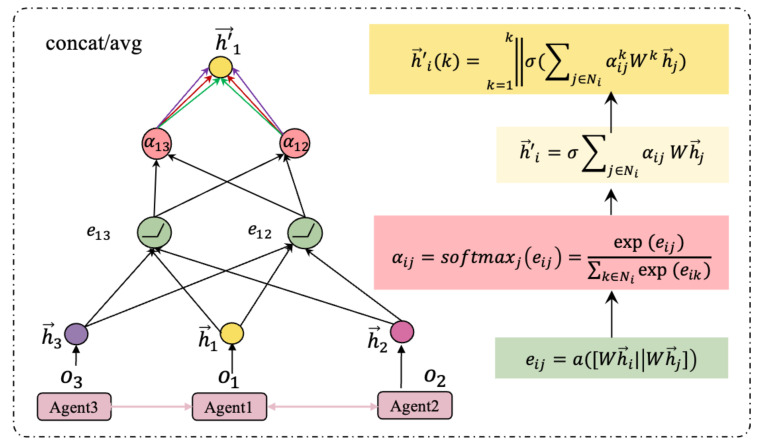
The information aggregation steps in GAT between connected agents. For agent 1, connected agent 2, and agent 3, calculate the attention weights of node 2: $\alpha_{12}=\frac{\exp(\mathrm{LeakyReLU}\left(e_{12}\right))}{\exp(\mathrm{LeakyReLU}\left(e_{12}\right))+\exp(\mathrm{LeakyReLU}\;\left(e_{13}\right))}$ and node 3: $\alpha_{13}=\frac{\exp(\mathrm{LeakyReLU}\left(e_{13}\right))}{\exp(\mathrm{LeakyReLU}\left(e_{12}\right))+\exp(\mathrm{LeakyReLU}\;\left(e_{13}\right))}$ towards node 1, respectively, to obtain the node embedding vector of agent 1 for a more robust state representation of the agent’s feature information.

Figure 2 illustrates the information aggregation steps of GAT. For target node *i*, calculate the similarity coefficients $e_{ij}$ between its neighbours $j\in N_{i}$ and itself.

(1) $$e_{ij}=a\left(\left[W{\vec{h}}_{i}∥W{\vec{h}}_{j}\right]\right),j\in N_{i}$$

A linear mapping with a shared parameter matrix *W* increases the dimension of vertex features, [.||.] concatenates the features after the transformation of node i, j, and a(.) maps concatenated high-dimensional features into a single real number using a single-layer feed-forward neural network. Then, the calculated attention coefficient $\alpha_{ij}$ is:

(2) $$\alpha_{ij}=\frac{\exp(LeakyReLU\left(e_{ij}\right))}{\sum_{z\in N_{i}}\exp\left(LeakyReLU\left(e_{iz}\right)\right)}$$

where $z\in N_{i}$ represents the other nodes that are connected to the current node *i*, except for node *j*. According to the attention weights, the new feature ${\vec{h}}_{i}^{\prime }$ of the target node *i*, after fusing the neighbourhood information, can be calculated by the feature weighted sum. In our study, we employ multi-head attention to measure the relevance interrelation among agents from different dimensions [29], enhancing the performance of GAT and the stability of the updated features, where *k* is the number of attention heads.

(3) $${\vec{h}}_{i}^{\prime }\left(k\right){=}_{k=1}^{k}∥\sigma \left(\sum_{j\in N_{i}}\alpha_{ij}^{k}W^{k}{\vec{h}}_{j}\right)$$

## 4. Methods

We propose a novel multi-agent hierarchical graph attention actor–critic reinforcement learning method (MAHGAC), as illustrated in Figure 1. MAHGAC employs the multi-agent actor–critic reinforcement learning network, in which agents interact with the environment to learn strategies through trial and error. The observation $o_{i}$ is encoded as a node embedding vector ${\vec{h^{\prime }}}_{i}$ through a hierarchical graph attention network, serving as an information-aggregated and contextualized state representation that adaptively captures the state dependencies among agents. This feature-embedding information is shared among all agents, allowing each agent *i* to receive contributions from other agents at each time step. After processing through a two-layer MPL, the ${\vec{h^{\prime }}}_{i}$ updates the action-value function $Q_{i}^{\psi }\left(o,a\right)=f_{i}\left(\left(o_{i},a_{i}\right),{\vec{h^{\prime }}}_{i}\right)$, guiding better collaboration among agents and promoting the learning of “advanced” strategies for complex interactive multi-agent tasks.

### 4.1. Agents Communication

**Figure 3:**
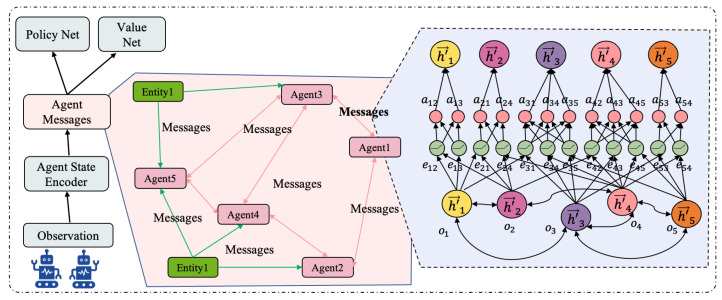
The information exchange process among agents. The connectivity diagram between agents, where nodes can exchange information through edges.

We model the multi-agent interaction as the graph G=(V,E), as shown in Figure 3. The entities (agents and landmarks) in the environment are abstracted as nodes n∈V on the graph, with edges e∈E between nodes that allow for communication. Agents exchange information and learn collaborative interactions along the edges of the graph. The observation of the agents is encoded as a node embedded vector, and the GAT (Section 3.2) weights the nodes connected to each agent to aggregate information effectively. The GAT encodes each agent’s local observations into a single node embedding vector, maintaining a constant dimensionality regardless of the number of agents. Thus, it generates a fixed-size environment representation. This stability allows for transferring learned policies to diverse scenarios, enhancing the model’s scalability.

In practice, agents who are close to each other have a greater influence on each other’s behaviour. We baked this critical information into the graph architecture, significantly enhancing the learning process. This approach provides a strong inductive bias for tasks with varying numbers of agents, enabling the model to perform exceptionally well in diverse and complex environments.

### 4.2. Hierarchical Graph Attention Network (HGAT)

As agents increase, information interactions in multi-agent systems grow increasingly complex. We propose the hierarchical graph attention network (HGAT), which enables agents to identify and prioritize critical interactive targets using “inter-agent” and “inter-group” attention layers. Thus, agents promote a deeper understanding of environmental information and make more effective decisions.

Step 1. Entities Clustering

**Figure 4:**
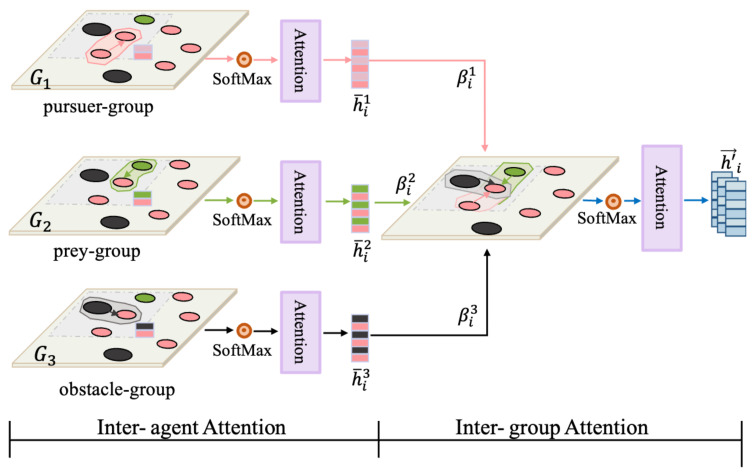
Hierarchical graph attention network. An example of the multi-agent pursuit task, where the entities in the environment are classified into 3 groups: pursuer-group, prey-group, and obstacle-group. In the “inter-agent” graph attention layer, attention weights are calculated between agents within each group, and then the aggregated feature vectors ${\vec{h}}_{i}^{g}$ are used as inputs for the “inter-group” graph attention layer to obtain information-aggregated and contextualized state representation ${\vec{h^{\prime }}}_{i}$.

We use prior knowledge or data to classify all of the entities in the environment (agents, landmarks, etc.) into different groups $C^{g}$. If it is a completely cooperative task, such as formation control, we classify all of the agents into one group. If it is cooperative navigation, we can cluster all of the agents into one group and the landmarks into another. If it is a mixed environment, such as a pursuit task, we can divide the pursuers into one group, the prey into another, and the obstacles into a separate group, as shown in Figure 4.

Step 2. “Inter-agent” Attention

The HGAT calculates the “inter-agent” attention and “inter-group” attention. Initially, through the node embedding vectors:

(4) $${\vec{h}}_{ij}^{g}=f_{ij}^{g}\left(s_{i},s_{j};W_{ij}^{g}\right)$$

where $i,j\in C^{g}\cap V\left(i\right)$ of neighbour nodes j∈V(i) adjacent to agent *i* in each group.

The embedding vector of the target node aggregation embedding ${\vec{h}}_{i}^{g}$ is calculated:

(5) $${\vec{h}}_{i}^{g}=\sum_{j\in C^{g}\cap V\left(i\right)}\alpha_{ij}^{g}{\vec{h}}_{ij}^{g}$$

The “inter-agent” attention weight $\alpha_{ij}^{g}$ quantifies the importance of the embedding ${\vec{h}}_{ij}^{g}$ from agent *j* to agent *i*, computed based on the GAT:

(6) $$\alpha_{i\cdot }^{g}\propto \exp\left(e_{i\cdot }^{g}\right)$$

where $e_{ij}^{g}=f_{\alpha }^{g}\left(s_{i},s_{j};W_{\alpha }^{g}\right)$. We employed multiple attention heads [29]; thus, the aggregated embedding of agent *i* is ${\vec{h}}_{i}^{g}$, where *k* is the number of attention heads.

(7) $${\vec{h}}_{i}^{g}=\prod_{k=1}^{K}\sum_{g=1}^{G}\sum_{j\in C^{g}\cap V\left(i\right)}\alpha_{ij}^{g}{\vec{h}}_{ij}^{g}$$

Step 3. “Inter-group” Attention

Then, HGAT computes the “inter-group” relationships, aggregates the group-level node embedding vector ${\vec{h}}_{i}^{1},\ldots ,{\vec{h}}_{i}^{g}$, and updates the feature-embedding vector ${\vec{h^{\prime }}}_{i}$ of agent *i* that incorporates contextual information.

(8) $${\vec{h^{\prime }}}_{i}=\sum_{g=1}^{G}\beta_{i}^{g}{\vec{h}}_{i}^{g}$$

where the “inter-group” attention weight $\beta_{i}$ measures the contribution of agent *i* across different subgroups, which guides which group agent *i* should focus on more to achieve its goal.

(9) $$\beta_{i}=\left(\beta_{i}^{1},\ldots ,\beta_{i}^{G}\right)\propto \exp\left(q_{i}\right)$$

(10) $$q_{i}=\left[q_{i}^{1},\ldots ,q_{i}^{G}\right]=f_{\beta }\left(\left[{\vec{h}}_{i}^{1},\ldots ,{\vec{h}}_{i}^{G}\right];W_{\beta }\right)$$

Agent *i* obtains an information-condensed and contextualized state representation ${\vec{h}}_{i}⟶{\vec{h^{\prime }}}_{i}$ using HGAT, which is particularly advantageous when addressing complex mixed cooperative–competitive multi-agent tasks. On the one hand, HGAT encodes the agent’s observations $o_{i}$ into node embedding vectors, where the dimensionality remains constant regardless of the number of agents, generating a fixed-size environment representation, thereby demonstrating scalability to larger tasks. On the other hand, by aggregating individual and group-level relationships, agents can discern the “role” they should play based on the relevance and importance of their contributions at each time step, enabling them to accomplish the final task more effectively.

### 4.3. Multi-Agent Actor-Critic

The node embedded feature vectors ${\vec{h^{\prime }}}_{i}$ are passed through a two-layer MLP $f_{i}$ and then input into the value and policy networks $Q_{i}^{\psi }\left(o,a\right)=f_{i}\left(g_{i}\left(a_{i},o_{i}\right),{\left\{{\vec{h^{\prime }}}_{j}\right\}}_{j\neq i}\right)$. These networks predict the estimated state value and the probability distribution of all possible actions, respectively. Each agent selects an action from this distribution, executes the chosen action, and receives a reward from the environment based on these actions. To promote exploration and reduce the risk of converging to suboptimal deterministic policies, we adopt the modern and widely recognized maximum entropy reinforcement learning approach to learn a soft value function [30]. This approach incorporates an entropy term into the policy gradient:

(11) $$\nabla_{\theta_{i}}J\left(\pi_{\theta }\right)=E_{s∼D,a∼\pi }\left[\nabla_{\theta_{i}}\log\left(\pi_{\theta_{i}}\left(a_{i}|o_{i}\right)\right)\left(-\alpha \log\left(\pi_{\theta_{i}}\left(a_{i}|o_{i}\right)\right)+Q_{i}^{\psi }\left(o,a\right)\right)\right]$$

Update all critics by minimizing a joint regression loss function through parameter sharing:

(12) $$L_{Q}\left(\psi \right)=\sum_{i=1}^{N}E_{(o,a,r,o^{\prime })∼D}\left[{\left(Q_{i}^{\psi }\left(o,a\right)-y_{i}\right)}^{2}\right]$$

(13) $$y_{i}=r_{i}+\gamma E_{a^{\prime }∼\pi_{\bar{\theta }}\left(o^{\prime }\right)}\left[Q_{i}^{\bar{\psi }}\left(o^{\prime },a^{\prime }\right)-\alpha \log\left(\pi_{{\bar{\theta }}_{i}}\left(a_{i}^{\prime }|o_{i}^{\prime }\right)\right)\right]$$

where $\bar{\psi }$ and $\bar{\theta }$ are the parameters of the target critics and target policies, respectively. $Q_{i}^{\psi }$ is the action-value estimate for agent *i*, which receives observations and actions for agents. α is the temperature parameter determining the balance between maximizing entropy and rewards.

### 4.4. MAHGAC Algorithm

The pseudocode for the MAHGAC method is depicted in Algorithm 1. We train using soft actor–critic, an off-policy actor–critic method for maximum entropy reinforcement learning [31]. During training, at each time point, generate a set of rollout, consisting of a tuple ${\left(o_{t},a_{t},r_{t},o_{t+1}\right)}_{1\ldots N}$, which is added to the replay buffer (1 × 10^6^). After an episode (25-time steps), reset the environment and perform 4 updates for the attention critic and all policies. For each update, we sample a mini-batch of 1024 time points from the replay buffer and then perform gradient descent on the Q−function loss and policy objective using an ADAM optimizer with a learning rate of 0.001 [32]. The agent encoder takes the 4-dim states as an input and outputs 128-dim embedding representations, and the encoders are a single ReLU fully connected (FC) layer. The communication module uses attention with 128-dim queries, keys, and values. Aggregated messages and the state of an agent are concatenated and updated by a single ReLU FC layer containing 128 neurons. We use K=3 communication hops between the agents.
**Algorithm 1** Training Procedure for MAHGAC  1:Initialize actor networks θ, critic network ψ  2:Initialize target networks $\bar{\psi }$ and $\bar{\theta }$, replay buffer *D*  3:**for** episode=1→M **do**  4:   Reset environments, and get initial $o_{i}^{e}$ for each agent *i*  5:   **for** t=1→T **do**  6:     Select actions $a_{i}∼\pi_{i}\left(\cdot |o_{i}\right)$ for each agent *i*  7:     Send actions to environment and get $o_{i}^{\prime },r_{i}$ for all agents  8:     Store transitions for all environments in *D*  9:     **for** g=1 to *G* for agent group $C^{g}$ **do**10:        **for** agent i=1 to $|C^{g}|$ in group $C^{g}$ **do**11:          Sample a minibatch $\left(o_{1\ldots N},a_{1\ldots N},r_{1\ldots N},{o^{\prime }}_{1\ldots N}\right)$12:          Calculate $Q_{i}^{\psi }\left(o_{1\ldots N},a_{1\ldots N}\right),a_{i}^{\prime }∼\pi_{i}^{\bar{\theta }}\left({\bar{o}}_{i}\right),Q_{i}^{\bar{\psi }}\left(o_{1\ldots N},a_{1\ldots N}\right)$13:          Set $y_{i}=r_{i}+\gamma E_{a^{\prime }∼\pi_{\bar{\theta }}\left(o^{\prime }\right)}\left[Q_{i}^{\bar{\psi }}\left(o^{\prime },a^{\prime }\right)-\alpha \log\left(\pi_{{\bar{\theta }}_{i}}\left(a_{i}^{\prime }|o_{i}^{\prime }\right)\right)\right]$14:          Update critic using $L_{Q}\left(\psi \right)=\sum_{i=1}^{N}E_{(o,a,r,o^{\prime })∼D}\left[{\left(Q_{i}^{\psi }\left(o,a\right)-y_{i}\right)}^{2}\right]$15:          Sample $\left(o_{1\ldots N}∼D\right)$16:          Calculate $a_{1\ldots N}∼\pi_{i}^{\bar{\theta }}\left(o_{i}^{\prime }\right),Q_{i}^{\psi }\left(o_{1\ldots N},a_{1\ldots N}\right)$17:          Update policies using:18:          $\nabla_{\theta_{i}}J\left(\pi_{\theta }\right)=E_{s∼D,a∼\pi }\left[\nabla_{\theta_{i}}\log\left(\pi_{\theta_{i}}\left(a_{i}|o_{i}\right)\right)\left(-\alpha \log\left(\pi_{\theta_{i}}\left(a_{i}|o_{i}\right)\right)+Q_{i}^{\psi }\left(o,a\right)\right)\right]$19:        **end for**20:     **end for**21:     Update target network parameters for each agent group $C^{g}$22:     ${\bar{\psi }}_{g}=\tau {\bar{\psi }}_{g}+\left(1-\tau \right)\psi_{g}$23:     ${\bar{\theta }}_{g}=\tau {\bar{\theta }}_{g}+\left(1-\tau \right)\theta_{g}$24:   **end for**25:**end for**

## 5. Experiments

### 5.1. Experimental Settings

We evaluate the effectiveness of the MAHGAC method in the multi-agent cooperative navigation task, multi-agent formation control task, and multi-agent confronting pursuit task. We exploit a multi-agent particle environment (MPE https://github.com/openai/maddpg, accessed on 11 September 2024) where agents can move in 2×2 sq. units 2D space. The action space for each agent is discretized, allowing agents to control unit acceleration or deceleration in the X and Y directions.

**Figure 5:**
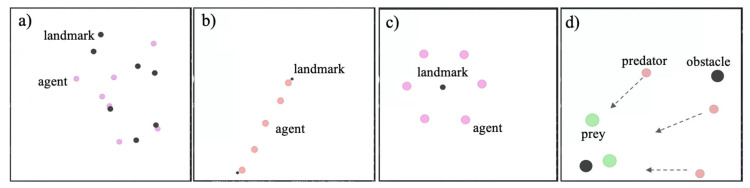
Experimental environments: (**a**) Cooperative navigation, where agents reach different landmarks while avoiding obstacles. (**b**) Linear formation, where agents form a line between two landmarks. (**c**) Regular polygon formation, where agents encircle landmarks to form a regular polygon. (**d**) Confronting pursuit, where pursuers collaborate to chase two prey, and when both prey are caught, the task is successful.

- **Cooperative navigation**: Figure 5a shows that the environment consists of *M* agents and *M* landmarks. The objective for each agent is to reach a distinct landmark while avoiding collisions with other agents. Each episode begins with *M* agents and *M* landmarks randomly initialised in the environment and ends after 25 time steps. During each episode, each agent receives a reward of −d based on its distance to the nearest landmark and incurs a penalty of −1 if it collides with another agent. Landmarks are not preassigned to agents, and agents dynamically determine which landmarks to target based on environmental feedback. Ultimately, each agent occupies a unique landmark, completing the navigation task and learning collaborative strategies.
- **Linear formation**: As shown in Figure 5b, there are *M* agents and two landmarks. The agents aim to position themselves equally spaced along a line between the two landmarks. Each episode begins with the agents and landmarks randomly initialized and ends after 25 time steps. Each agent receives a reward of −d based on the distance between its current position and the expected position along the line.
- **Regular polygonal formation**: As shown in Figure 5c, there are *M* agents and one landmark. The agents are required to position themselves into an *M*-sided regular polygonal formation with the landmark at its centre. Each episode starts with the agents, and the landmark randomly initializes and ends after 25 time steps. During the episode, each agent receives a reward of −d based on the distance between its current position relative to the landmark and the expected position in the polygonal formation.
- **Confronting pursuit**: Figure 5d shows that the environment consists of *M* pursuers and *N* prey. The competitive game objective is that the *M* homogeneous pursuers pursue the *N* prey while the prey strives to escape. As pursuers have lower speed and acceleration compared with prey, they must cooperate effectively to succeed in their pursuit. Each pursuer obtains a positive reward of +10 when it catches the prey, while the prey incurs a negative reward of −10. To prevent prey from straying too far from a designated zone, they receive a negative reward if they leave this area. The environment also contains obstacles, and any agent colliding with an obstacle is penalized with a negative reward of −10.

In our work, we conducted a series of comparative experiments to evaluate the performance of different MARL methods across various tasks. We selected four methods for comparison: MADDPG [33] without attention, G2ANet [26] with attention, DGN [27] with single-layer graph attention, and MAAC [24] with actor–attention–critic. All methods were tested under identical experimental and training settings. Each experiment was conducted five times, and the results were reported using average values and standard deviations to describe the range of outcomes. To comprehensively evaluate their performance, we employed two main evaluation metrics:

- Success rate (S%): percentage of tasks completed during evaluation episodes (higher is better).
- Mean episode length (MEL): average length of successful episodes during evaluation (lower is better).

### 5.2. Results

#### 5.2.1. Effectiveness

**Figure 6:**
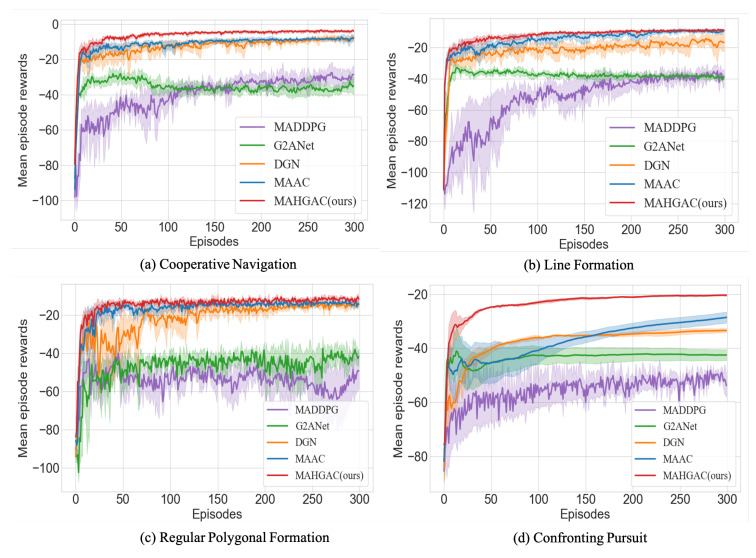
Subfigure (**a**) represents the mean episode rewards curves of 3 agents in the cooperative navigation task. (**b**) represents the mean episode rewards curves of 5 agents in the linear formation task. (**c**) represents the mean episode rewards curves of 4 agents in the regular polygonal formation task. (**d**) represents the mean episode rewards curves for the task of 3 pursuers cooperating to pursue 2 prey.

Figure 6 illustrates the mean episode reward curves of each method across four tasks. Each curve represents the average results of multiple experiments, with the shaded area indicating the standard deviation. In both fully cooperative and mixed cooperative–competitive tasks, the reward curves of MAHGAC converge to higher levels, demonstrating superior performance compared with other methods. Additionally, MAHGAC outperforms other methods employing single-layer graph attention in mixed cooperative–competitive tasks. This advantage is attributed to the increased complexity of agent relationships, which demand more interactions and sophisticated information selection. MAHGAC adaptively extracts state-dependent relationships among multiple agents, enhancing information selection and strategy learning.

Table 1 presents the success rate (S%) and mean episode length (MEL) for each method in both fully cooperative and mixed cooperative–competitive tasks. Compared with MADDPG without attention, MAHGAC significantly outperforms in success rate across all tasks, while MEL remains consistent. Compared with G2ANet with attention, MAHGAC achieves higher success rates across all tasks and maintains lower MEL in cooperative navigation, linear formation, and pursuit tasks.

In fully cooperative tasks, MAHGAC demonstrates an average success rate improvement of 8.054% over DGN with single-layer graph attention, with the MEL being reduced by an average of 0.4. Compared with MAAC with actor–attention–critic, MAHGAC demonstrates an average success rate improvement of 0.98% and a reduction in MEL by an average of 0.316.

In mixed cooperative–competitive tasks, MAHGAC improves average success rates by 19.942% and decreases MEL by 0.58 on average. Compared with MAAC, MAHGAC achieves an average success rate increase of 7.961% and an MEL reduction of 1.09 on average. MAHGAC demonstrates significant advantages in complex, fully cooperative tasks and mixed cooperative–competitive tasks, particularly those with abundant information.

Notably, MAHGAC surpasses other graph attention methods in handling complex agent interactions. This improvement is attributed to the HGAT mechanism, which models state dependencies at both individual agent and subgroup levels. The “inter-agent” graph attention layer effectively captures interactions within each subgroup, enabling agents to adjust their role positioning. The “inter-group” graph attention layer helps agents learn different subgroups’ relationships, enhancing their ability to adapt to dynamic environments involving opponents, teammates, or obstacles. The HGAT mechanism provides agents with a clearer understanding of their roles (“Who am I?”) and actions (“What should I do at each time point?”).

#### 5.2.2. Scalability

**Figure 7:**
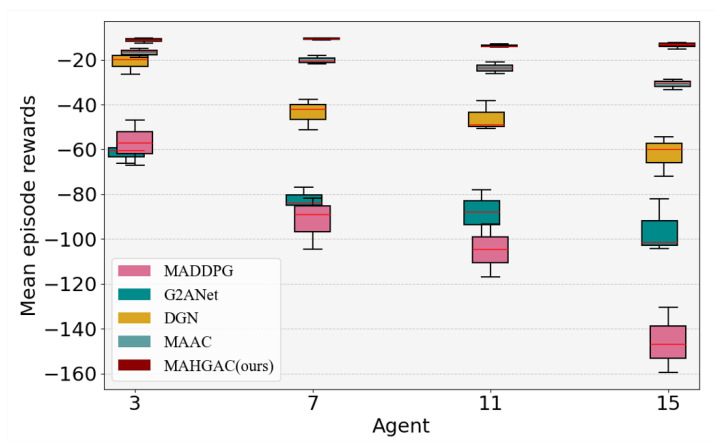
The average episode rewards of different methods in cooperative navigation task with different numbers of agents.

As shown in Figure 7, we compare the average episode rewards for the cooperative navigation task with different numbers of agents. As the number of agents and task complexity increase, interactions between agents become more complex, resulting in a sharp decline in the MADDPG rewards, indicating its limited scalability. Similarly, G2ANet, DGN, and MAAC show a decline in reward performance as agent numbers increase, albeit to different extents. In contrast, when the number of agents increases from 3 to 15, MAHGAC remains stable performance, demonstrating its scalability. Furthermore, the boxplot shapes reveal that MADDPG, G2ANet, and DGN exhibit relatively scattered convergence values across multiple tests. At the same time, MAHGAC and MAAC display more consistent convergence values, further indicating the generalization capability.

Table 2 shows the success rate for the cooperative navigation task with different numbers of agents. When the number of agents is three, all methods complete the task. However, as the number of agents increases to seven, both MADDPG and G2ANet struggle, whereas the success rate of MAHGAC improves by 16.07% compared with DGN with single-layer graph attention. As the number of agents increases to 11 and 15, MADDPG and G2ANet fail to complete the task, while DGN, MAAC, and MAHGAC can still be successful.

Moreover, compared with DGN with single-layer graph attention, the success rate of MAHGAC increases by 33.66% and 38.075% at N = 11 and N = 15, respectively. When compared with MAAC with actor–attention–critic, the success rate of MAHGAC increases by 4.343% and 6.074% at N = 11 and N = 15, respectively. As the number of agents increases, MAHGAC demonstrates exceptional stability, with a success rate standard deviation of 0.093, compared with 12.618 for DGN and 1.922 for MAAC. The trends in the boxplot in Figure 7 further affirm the superior stability of the MAHGAC. MAHGAC maintains its performance with increasing agent numbers, showcasing robust scalability and remarkable stability.

## 6. Curriculum Learning

As the number and dimensions of agents increase, training the model from scratch becomes increasingly time-consuming, leading to slower convergence rates. To address this challenge, we adopted the curriculum learning approach to facilitate model transferability. Curriculum learning is a training strategy that emulates the human learning process, advocating that models start learning from simple samples and progressively transition to more complex samples and tasks [34].

In this work, we conducted curriculum learning experiments by training our strategy within this framework and using a line formation task for verification. Notably, the purpose of this experiment was solely to verify that the MAHGAC method could be effectively transferred using curriculum learning.

In the MAHGAC, network parameters are shared between agents, facilitating the direct application of the policy π. Initially trained for a task *Q* with *N* agents, this policy can subsequently be fine-tuned to perform different tasks $Q^{\prime }$ with *M* agents. We trained on a line formation task with five agents. Upon reaching an 85% success rate, and we transferred the learned strategy to a 10-agent team.

**Figure 8:**
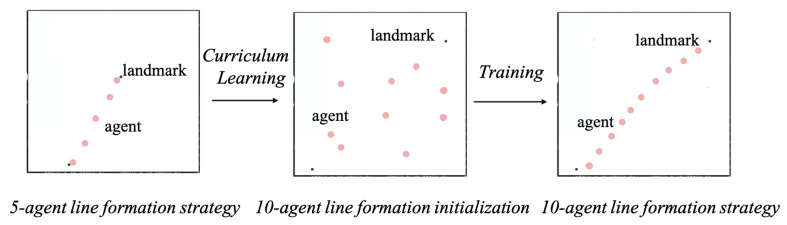
The 5-agent line formation strategy is transferred to a new 10-agent line formation task through curriculum learning.

Figure 8 illustrates transferring the 5-agent line formation strategy to a new 10-agent team using curriculum learning. As shown in Table 3, the 10-agent team successfully learned the cooperative strategy, achieving a 90.51% success rate and 21.82 MEL with curriculum learning, demonstrating a 2.89% improvement in success rate and a 3.33 reduction in MEL. This comparison underscores the effectiveness of curriculum learning in accelerating and enhancing the completion of line formation tasks.

## 7. Conclusions

We propose an innovative multi-agent hierarchical graph attention actor–critic reinforcement learning method (MAHGAC) that leverages graph attention networks to encode agent’s observations into a single node embedding vector, maintaining a constant dimensionality regardless of the number of agents, generating a fixed-size environment representation, offering flexibility and scalability. We introduce a hierarchical graph attention mechanism (HGAT) to further capture complex interactions. HGAT employs “inter-agent” and “inter-group” graph attention layers to update agents’ observations into information-condensed and contextualized state representations. Compared with other attention communication, HGAT utilizes the topological structure of the graph, allowing for more flexible handling of high-dimensional agent information, effectively models state dependencies at individual and group levels, enabling agents to focus on interactions with the most relevant objects and learn more sophisticated strategies.

We conducted experiments to evaluate MAHGAC’s effectiveness and scalability. Compared with baseline methods, MAHGAC demonstrates stability performance and superior scalability in both fully cooperative and mixed cooperative–competitive scenarios. Furthermore, we evaluated MAHGAC’s transferability within a curriculum learning framework in the linear formation task. Experimental results show that MAHGAC not only sustains performance as the task complex but also exhibits superior stability, scalability, and transferability, offering new possibilities for addressing larger-scale tasks in practice.

## Figures and Tables

**Table 1:** Success rate (S%) and mean episode length (MEL) of different methods in different tasks.

Method	Cooperative Navigation (N = 3)		Linear Formation (N = 5)		Regular Polygonal (N = 4)		Pursuit (N = 4)	
	S (%)	MEL	S (%)	MEL	S (%)	MEL	S (%)	MEL
MADDPG	47.389	3.86	55.938	7.12	65.994	5.54	53.935	5.84
G2ANet	54.983	5.04	61.214	8.22	70.186	5.37	58.420	6.77
DGN	79.083	4.10	79.617	7.70	87.710	5.53	66.708	6.30
MAAC	85.025	4.39	90.720	7.23	91.883	5.45	78.689	6.87
MAHGAC	86.378	3.72	91.330	7.11	92.865	5.29	86.650	5.78

**Table 2:** Success rates (S%) for the cooperative navigation task.

Method	Cooperative Navigation (S%)			
	N = 3	N = 7	N = 11	N = 15
MADDPG	47.389	13.850	-	-
G2ANet	54.987	28.076	-	-
DGN	79.083	70.200	52.775	48.120
MAAC	85.025	83.883	82.092	80.121
MAHGAC	86.378	86.272	86.435	86.195

**Table 3:** Curriculum learning for line formation.

Normal Training				Curriculum Learning Training			
N = 0		N = 10		N = 5		N = 10	
**S (%)**	**MEL**	**S (%)**	**MEL**	**S (%)**	**MEL**	**S (%)**	**MEL**
0	0	87.62	21.82	91.33	7.11	90.513	18.49

## Data Availability

Data is contained within the article.
